# Peritonsillar Abscess and Post-aspiration Bleed Identified with Point-of-care Ultrasound Using Endocavitary Probe: A Case Report

**DOI:** 10.5811/cpcem.1645

**Published:** 2024-03-26

**Authors:** Jaclyn Floyd, Brandon Dahl, Matthew Whited, Ami Kurzweil

**Affiliations:** Eisenhower Medical Center, Department of Emergency Medicine, Rancho Mirage, California

**Keywords:** *peritonsillar abscess*, *endocavitary ultrasound probe*, *ultrasound*, *otolaryngology*, *case report*

## Abstract

**Introduction:**

Peritonsillar abscesses form between the tonsillar capsule, the superior constrictor, and palatopharyngeus muscles. Physicians traditionally make this diagnosis clinically; however, ultrasound allows clinicians to further identify and differentiate between peritonsillitis, peritonsillar abscess, and phlegmon formation. By increasing both the sensitivity and specificity, ultrasound improves the diagnostic accuracy for patients with peritonsillar abscesses. This case demonstrates the utilization of ultrasound in peritonsillar abscesses and the application of point-of-care ultrasound (POCUS) in identifying complications of procedures used for treatment in the emergency department (ED).

**Case Report:**

A 19-year-old male presented to the ED with complaints of severe sore throat and fever for the prior five days. A POCUS using an endocavitary probe with sterile cover demonstrated hypoechoic debris with a “swirl sign.” Ultrasound was used to successfully guide needle aspiration by using in-plane needle guidance. The patient had significant bleeding after needle aspiration, and repeat POCUS clearly identified a new pocket of blood that had formed and was contained in the soft tissue. We monitored the size of the hematoma in real time with ultrasound to ensure the hematoma had no rapid expansion and was stable.

**Conclusion:**

Among the differential diagnoses for sore throat, the diagnosis of peritonsillar abscess is particularly concerning as it is both common and generally requires swift intervention. Presentations can range from a mild infection to a life-threatening emergency with potential airway compromise. The two primary avenues for treatment include either needle aspiration or incision and drainage. Ultrasound can successfully identify the abscess and other landmarks for safe and successful drainage, as well as early identification of complications.

Population Health Research Capsule What do we already know about this clinical entity?

*Peritonsillar abscess is the most common deep-space infection of the head and neck.*

What makes this presentation of disease reportable?

*This case highlights the rapid onset of a hematoma following needle aspiration and the use of ultrasound to monitor its progression.*

What is the major learning point?

*Hematoma development may be one cause of bleeding after needle aspiration of peritonsillar abscess and this can be seen with a point-of-care ultrasound.*

How might this improve emergency medicine practice?

*Clinicians can utilize ultrasound guidance to help manage complications of needle aspirations for peritonsillar abscess, with an image and video for reference.*

## INTRODUCTION

Peritonsillar abscess is the most common deep-space infection of the head and neck. Cases are commonly polymicrobial but most commonly caused by streptococcus. Patients may present with fever, odynophagia, dysphagia, trismus and possibly a muffled voice colloquially known as “hot potato voice.” Diagnosis of peritonsillar abscess using history and physical alone has a sensitivity and specificity of 75% and 50%, respectively. Diagnosis using ultrasound, in addition to history and physical, using an endocavitary probe placed intraorally showed a sensitivity and specificity of 91% and 75%, respectively, while transcervical showed a sensitivity and specificity of 80% and 81%, respectively.^7^ This case highlights an interesting complication of acute bleed demonstrated by point-of-care ultrasound (POCUS) during peritonsillar drainage. Ultrasound identification of peritonsillar abscess is on the consensus list for competencies in emergency medicine residency^3^; proficiency in ultrasound is increasingly expected in trainees, and this particular scenario exemplifies its utility.

## CASE PRESENTATION

A 19-year-old male with no past medical history presented to the emergency department (ED) with the complaint of six days of sore throat, primarily on the right side. He also had fever and chills, otalgia, and odynophagia. On physical exam the patient’s voice had a muffled tone, he felt warm to touch, and there was swelling next to the right tonsil with uvular deviation. He was not having any difficulty breathing, and no stridor was noted. Computed tomography (CT) had been ordered, which returned showing a right peritonsillar abscess measuring 23 × 32 × 45 millimeters, enlargement of Waldeyer’s ring, and right-sided level II and III lymphadenopathy. A POCUS using the endocavitary probe with sterile cover demonstrated encapsulated, swirling echogenic debris approximately 3 centimeters (cm) deep. This was used to clearly mark the location for needle aspiration.

Needle aspiration was performed with removal of 10 milliliters (mL) of purulence, and the patient started to have significant bleeding, approximately 200 milliliters of both blood and saliva in the suction cannister within a few minutes. A solution of 4% cocaine was soaked onto long cotton tips, and pressure was held for 10 minutes. Repeat ultrasound was performed every two minutes, which showed a hyperechoic pocket, likely representing fresh hematoma, not expanding. Two physicians were in the room, with one physician continuing to hold pressure, although moving slightly laterally at each two-minute interval to allow space for the second physician to perform a repeat ultrasound. Each repeat ultrasound demonstrated stability of the hematoma, and at 10 minutes the hematoma was considered controlled. Clinically at this point, the patient had no further oropharyngeal bleeding.

Otolaryngology was consulted regarding the findings and recommended additional incision and drainage (I&D) given the initial size of the abscess found on CT. In addition, they recommended intravenous (IV) antibiotics, IV dexamethasone, soft diet, and admission. The I&D was performed in the operating room, and the patient was discharged the following day on clindamycin, orally and a medrol dose pack.

## DISCUSSION

Peritonsillar abscess is a frequently made diagnosis both in urgent care and in the ED. It affects an estimated 45,000 people per year, with over half being admitted for further treatment.^10^ Physical exam will reveal a unilateral (rarely bilateral) swelling above and lateral to the tonsils with contralateral deviation of the uvula. Peritonsillar abscess is a medical emergency due to the possibility of upper airway obstruction and the patient’s inability to protect their own airway. Diagnosis is clinical; however, ultrasound can be used to differentiate between peritonsillitis and peritonsillar abscess. Treatment includes I&D or needle aspiration and antibiotics.

Ultrasound can be used to perform I&D or needle aspiration and has been shown helpful in identifying the location of the abscess and increasing the safety of the procedure by aiding in identifying arterial vasculature such as the underlying carotid artery and its distance from the abscess pocket (Image). While ultrasound can help minimize complications, they can still occur. The most frequent complications of peritonsillar abscess include mediastinitis, necrotizing fasciitis, Lemierre syndrome, and retropharyngeal abscess.^2^ Antibiotic treatment should cover staphylococcus and streptococcus, anaerobes, *Eikenella corrodens,* and *Haemophilus influenzae*. Intravenous antibiotic options include ampicillin/sulbactam, piperacillin/tazobactam, clindamycin or ceftriaxone. Outpatient antibiotic options include clindamycin or amoxicillin/clavulanate.

**Image.**
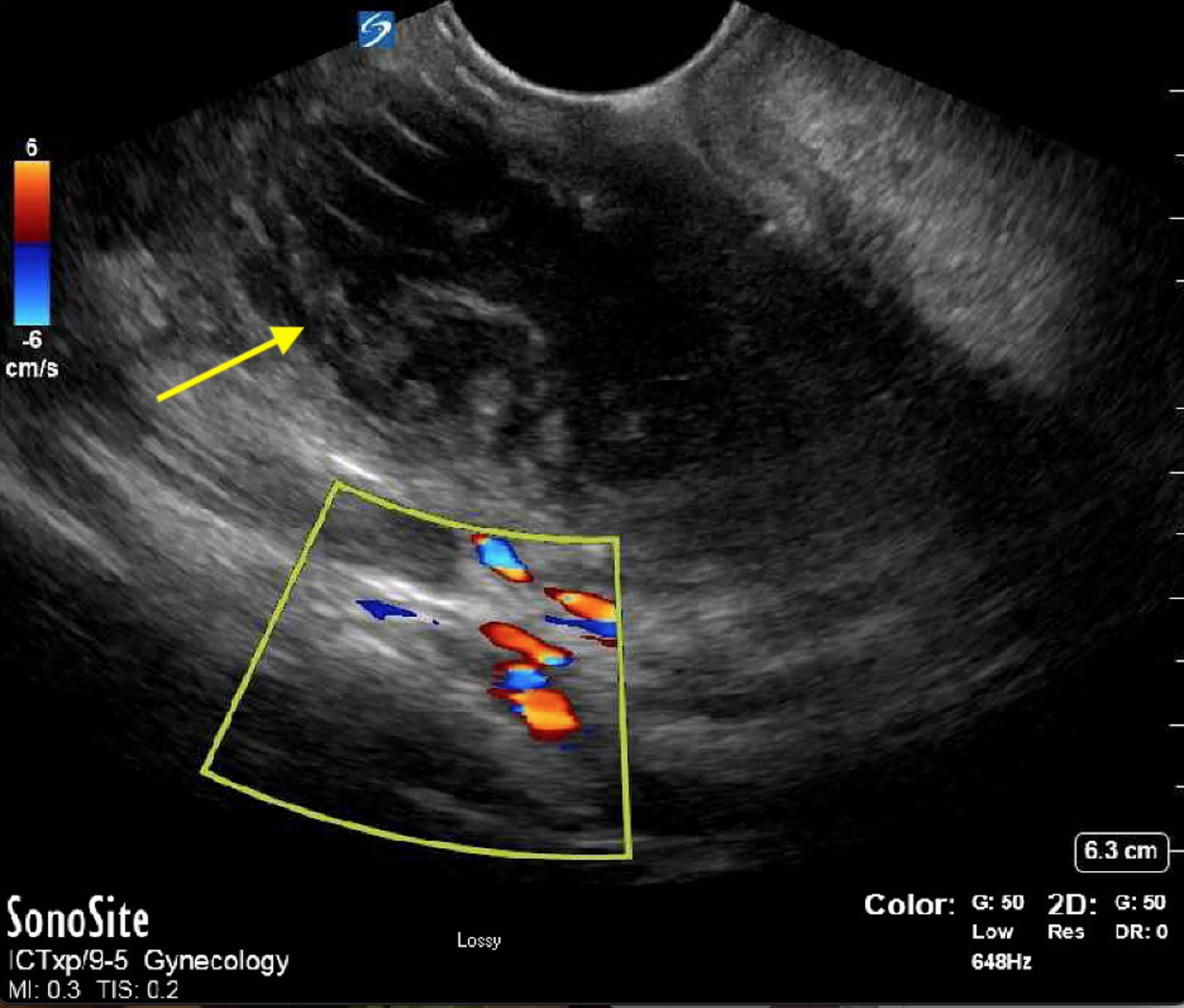
Point-of-care ultrasound with endocavitary probe demonstrating swirling echogenic debris encapsulated approximately 3 centimeters deep, representing initial abscess (arrow). Color box represents vasculature, including carotid artery.

Ultrasound can be helpful in identifying crucial aspects of each of these complications. For example, in necrotizing fasciitis ultrasound has a high sensitivity in identifying free air.^9^ For retropharyngeal abscess, ultrasound can identify the pocket of fluid, a utilization that has mostly been described in pediatrics.^11^ This case shows an unexpected complication of needle aspiration. The hematoma shown in Video was monitored using the endocavitary probe to ensure it was not rapidly expanding as pressure was held on the cavity exteriorly. Pressure was held continuously for 10 minutes, while the endocavitary probe was used every two minutes to monitor the hematoma. This case exemplifies how the endocavitary probe approach can be extremely useful in helping to manage a peritonsillar abscess as well as complications that may arise.

## CONCLUSION

The skill of using ultrasound to detect peritonsillar abscess is acknowledged as a key competency for emergency medicine residents.^3^ This case study demonstrates the utility of point-of-care ultrasound in both identifying peritonsillar abscesses and providing guidance during the aspiration procedure. Gaining a visual diagnosis when complications arise can prove to be exceptionally valuable.

## Supplementary Information

Video. Point-of-care ultrasound demonstrating hyperechoic pocket, representing fresh hematoma (arrow).
